# The Effect of Oxcarbazepine on the Pharmacokinetics of Single-Dose Aripiprazole in a Rat Model

**DOI:** 10.3390/medicines13020013

**Published:** 2026-04-01

**Authors:** Iulia-Maria Ciocotișan, Dana Maria Muntean, Laurian Vlase

**Affiliations:** Department of Pharmaceutical Technology and Biopharmacy, “Iuliu Hațieganu” University of Medicine and Pharmacy, Victor Babeș Street 41, 400012 Cluj-Napoca, Romania; iulia.ciocotisan@umfcluj.ro (I.-M.C.); laurian.vlase@umfcluj.ro (L.V.)

**Keywords:** pharmacokinetics, drug interactions, aripiprazole, dehydroaripiprazole, oxcarbazepine

## Abstract

**Background/Objectives**: A possible pharmacokinetic interaction between a single dose of aripiprazole and multiple-dose pretreatment with oxcarbazepine was investigated in vivo in Wistar albino rats. **Methods**: The experiment was conducted on two groups of 12 male rats each. The control group received a single oral dose of aripiprazole (8 mg/kg), while the test group was given oral oxcarbazepine (85 mg/kg/day) for 5 days, followed by a single oral dose of aripiprazole (8 mg/kg). Blood samples were automatically drawn following the administration of aripiprazole to each rat. Noncompartmental analysis was employed to determine the pharmacokinetic parameters of aripiprazole and its active metabolite, dehydroaripiprazole. **Results**: After the five-day oxcarbazepine pretreatment, aripiprazole’s maximum plasma concentration decreased by 51.37%, and its mean half-life was significantly reduced by 1.51-fold. In contrast, for the metabolite, the mean total area under the concentration–time curve increased by 44.66%, and the mean apparent systemic clearance decreased by 61.84%. **Conclusions**: Multiple-dose pretreatment with oxcarbazepine resulted in significant changes in the pharmacokinetics of a single oral dose of aripiprazole and its active metabolite in vivo in rats. The clinical implications should be further studied in human subjects, as this interaction may reduce the efficacy of aripiprazole.

## 1. Introduction

Aripiprazole (ARI), a quinolinone derivative, is a second-generation antipsychotic with partial agonist activity at dopamine D_2_ receptors, including presynaptic D_2_ autoreceptors. Its activity at dopamine D_2_, D_3_ and serotonin 5-HT_1A_ receptors, along with 5-HT_2A_ receptor antagonism, makes it effective in reducing the positive, negative and cognitive symptoms of schizophrenia, with fewer side effects such as movement disorders and weight gain compared to older antipsychotics [1]. Other approved indications include bipolar I disorder, adjunctive treatment of major depressive disorder, irritability associated with autistic disorder and treatment of Tourette’s syndrome [2,3]. It is also used off-label for conditions such as anxiety disorders, personality disorders and agitation, either in pediatric populations or associated with dementia [4]. ARI undergoes phase I metabolic reactions (via hepatic cytochrome P450 (CYP) enzymes), including dehydrogenation and hydroxylation mediated by CYP3A4 and CYP2D6, and N-dealkylation mediated by CYP3A4. These enzymes are responsible for the formation and further metabolism of the active metabolite dehydroaripiprazole (D-ARI), which has similar pharmacological properties to ARI and accounts for about 40% of the active moiety of ARI [5]. This is why the sum of the parent drug and D-ARI is often used in therapeutic measurement [6]. The average elimination half-life is approximately 75 h for ARI and 95 h for D-ARI at steady state in extensive metabolizers. ARI exhibits linear pharmacokinetics and 87% oral bioavailability, reaches maximum plasma concentration (C_max_) in 3–5 h and achieves steady state within 2 weeks of daily intake [1].

Oxcarbazepine (OXC), a dibenzoazepine derivative and 10-keto analog of carbamazepine, is an antiseizure drug approved for monotherapy or adjunctive treatment of partial-onset seizures in children and adults [7]. Additionally, it is commonly prescribed as a mood stabilizer. Cytosolic aryl-ketone reductases rapidly and completely reduce OXC to its active metabolite, 10,11-dihydro-10-hydroxycarbazepine (licarbazepine), a racemic mixture of eslicarbazepine and (R)-licarbazepine. Since the metabolite is responsible for the therapeutic effect by blocking voltage-gated sodium channels, OXC is considered a prodrug [8]. OXC is a dose-dependent, mild-to-moderate inducer of the CYP3A4 isoenzyme. At daily doses of 1200 mg or higher, the risk of interaction via this metabolic pathway may become clinically relevant [9].

In certain patients, the coadministration of ARI and OXC may be necessary. Up to 50% of epilepsy patients have psychiatric or neurologic comorbidities, with mood disorders being among the most prevalent. In this regard, both ARI and OXC can be prescribed in bipolar disorders. Moreover, patients with epilepsy have an increased risk of developing mood, anxiety and psychotic disorders. Conversely, individuals with these psychiatric disorders also have a greater risk of developing epilepsy. This highlights the bidirectional relationship between epilepsy and psychiatric disorders [10,11]. However, to date, no pharmacokinetic studies have specifically addressed the potential interaction between ARI and OXC. Only a few case reports have been published on this topic, underscoring the need for pharmacokinetic evaluation to better understand the potential for drug–drug interactions between these two psychopharmacological agents. Enzyme-inducing antiseizure drugs can accelerate the clearance of psychotropic drugs metabolized in the liver, such as ARI, potentially reducing their efficacy. Since OXC induces the CYP3A4 isoenzyme and ARI is a substrate of the same enzyme, decreased blood concentrations of ARI are likely. OXC’s potential for clinically relevant interactions, despite its perceived safety compared to carbamazepine, should not be overlooked [12].

The study aimed to assess the pharmacokinetic effects of a five-day pretreatment with OXC on a single dose of ARI, in vivo, using an animal model.

## 2. Materials and Methods

### 2.1. Chemicals and Reagents

Aripiprazole used for rat administration was sourced from Astoret^®^ 10 mg orodispersible tablets (Terapia, Cluj-Napoca, Romania); dehydroaripiprazole analytical standard (99.66% purity) from MedChemExpress (South Brunswick, NJ, USA); and oxcarbazepine from Trileptal^®^ 600 mg film-coated tablets (Novartis, Nuremberg, Germany). Aripiprazole pharmaceutical primary standard (purity ≥ 99%), haloperidol pharmaceutical primary standard, 98% formic acid and acetonitrile (analytical grade) were acquired from Merck (Darmstadt, Germany). Sodium Heparin 5000 IU/mL (Belmedpreparaty, Minsk, Belarus), ketamine (Vetased^®^ 10%, Farmavet, Bucharest, Romania), xylazine (XylazinBio^®^ 2%, Bioveta, Ivanovice na Hané, Czech Republic) and saline solution (NaCl 0.9%, B Braun, Melsungen, Germany) were also used.

### 2.2. Animals

Twenty-four healthy adult (8–14 weeks, mean ± SD: 10.25 ± 2.42 weeks) male Wistar rats (*Rattus norvegicus*, albino strain) from the Centre for Experimental Medicine and Practical Skills (“Iuliu Hatieganu” University of Medicine and Pharmacy, Cluj-Napoca, Romania), weighing 230–390 g (mean ± SD: 300 ± 52.6 g), were obtained. Standard conditions ensured animal well-being: temperature 20–26 °C, relative humidity 30–70%, 12 h light–dark cycles, proper ventilation, minimal noise, adequate cage size and clean wood shavings. They had continuous access to clean water and pellet food.

### 2.3. Experimental Procedures

The 24 rats were assigned to a test group (*n* = 12) receiving daily oral doses of OXC 85 mg/kg for five days, followed by a single oral dose of ARI 8 mg/kg administered 30 min after the last OXC dose, and to a control group (*n* = 12) receiving a single oral dose of ARI 8 mg/kg. Following each ARI administration in both experimental groups, 18 blood samples (100 µL each) were collected per rat. One day before blood collection and ARI administration, the rats underwent femoral vein cannulation under ketamine:xylazine (1:1, *v*/*v*) anesthesia (0.1 mL/100 g body weight (b.w.)) at a dose of 50 mg/kg ketamine and 10 mg/kg xylazine, administered intramuscularly, to allow direct vascular access for automatic pharmacokinetic sampling This dosage falls within the recommended range for rodent anesthesia, which lists ketamine (40–90 mg/kg) and xylazine (5–10 mg/kg) as a preferred injectable anesthetic combination for rats [13]. The intramuscular route was chosen to ensure a faster onset of anesthesia and reduce variability in absorption compared to the intraperitoneal route. The duration of anesthesia achieved was sufficient for the surgical procedure, and the relatively shorter duration was advantageous for minimizing recovery time. The day following surgery, the rats were connected to the BASi Culex ABC^®^ Automatic Blood Collector device (BASi, West Lafayette, IN, USA). Blood samples were collected in heparinized tubes at scheduled times (10, 20, 30, 45 min, 1, 1.5, 2, 2.5, 3, 4, 6, 8, 10, 12, 16, 20, 24 and 30 h) after each ARI administration and stored at −20 °C. To avoid fluid depletion, the excess blood collected on the catheter of the device during sampling was reinfused into the rat, along with a volume of saline and heparin mixture (1 mL 5000 IU heparin to 500 mL saline) equal to the volume of blood collected.

The OXC dose of 85 mg/kg was selected based on a typical human therapeutic dose of 600 mg/day. Assuming an average adult body weight of 70 kg, this corresponds to approximately 8.5 mg/kg. Although the FDA-recommended body surface area scaling factor for converting human doses to rats is 6.2 [14], we applied a higher scaling factor of 10 to account for the increased metabolic rate and hepatic enzyme activity in rats. This yielded a rat dose of OXC of 85 mg/kg. Steady state is typically achieved within 2–3 days of twice-daily OXC administration [7]. Because antiepileptic drugs generally exhibit shorter half-lives in rodents compared to humans, we assumed steady-state exposure was reached within the 5-day pretreatment period in rats [15]. Similarly, a dose of 8 mg/kg b.w. of ARI was selected based on the human therapeutic range of 15–30 mg. Applying a 10-fold scaling factor yielded a maximum rat dose of ~4.3 mg/kg. However, to ensure sufficient systemic exposure for pharmacokinetic profiling and to accommodate the sensitivity of the analytical method, the ARI dose was increased to 8 mg/kg.

ARI and OXC suspensions were prepared from commercially available tablets, Astoret^®^ 10 mg and Trileptal^®^ 600 mg, respectively. Each formulation was finely ground using a mortar and pestle, and the resulting powder was suspended in 1% (*w*/*v*) carboxymethyl cellulose aqueous dispersion without separation of excipients. The prepared suspensions were vortexed thoroughly before each administration to ensure homogeneity and were immediately drawn into oral gavage syringes and administered to the animals.

### 2.4. Analytical Method

Thawed blood samples were spiked with haloperidol as an internal standard. Each 100 µL blood sample was treated with 300 µL acetonitrile for protein precipitation, vortexed for 10 s using IKA Vortex 2 at 1000 rpm (IKA®-Werke GmbH & Co., KG, Staufen im Breisgau, Deutschland)) and centrifuged for 5 min at 10,000 rpm with Sigma 3-30KS centrifuge (Sigma Laborzentrifugen GmbH, Osterode am Harz, Germany), 9168× *g*. Supernatants were transferred to autosampler vials for ARI and D-ARI quantification. A previously validated and described LC-MS/MS method using an Agilent 1100 HPLC system with a binary pump, autosampler and thermostat (Agilent Technologies, Santa Clara, CA, USA) was used for quantifying ARI and D-ARI. Briefly, the method demonstrated acceptable sensitivity, precision, accuracy, matrix effects and analyte recovery in accordance with regulatory guidelines [16]. The analytes were separated chromatographically using a Zorbax SB-C18 column (100 × 3.0 mm, 3.5 µm) (Agilent Technologies, Santa Clara, CA, USA) and detected with the Bruker Ion Trap SL (Bruker Daltonics GmbH, Bremen, Germany). The mobile phase, consisting of 0.3% (*m*/*v*) formic acid in water and acetonitrile, was delivered in a linear gradient: 20% acetonitrile, increasing to 38% acetonitrile at 2.5 min, maintained at 38% until 4.2 min, then re-equilibrated with 20% acetonitrile for 2 min. The flow rate was 1 mL/min, the volume injected was 5 µL and the column temperature was set at 40 °C. Positive ions were generated using an electrospray ionization source, and mass spectrometry detection was set to multiple reaction monitoring mode. The ion transitions monitored were *m*/*z* 448 → *m*/*z* 285 (ARI), *m*/*z* 446 → *m*/*z* 285 (D-ARI) and *m*/*z* 376 → *m*/*z* 165 (haloperidol). The retention times were 3.55 min (ARI), 3.15 min (D-ARI) and 2.80 min (haloperidol). The calibration curves for both ARI and D-ARI, prepared using a dehydroaripiprazole analytical standard (99.66% purity) and an aripiprazole pharmaceutical primary standard (purity ≥ 99%), were linear between 2 and 50 ng/mL.

### 2.5. Data Analysis

Phoenix WinNonlin software version 8.5.2 (Pharsight Company, Mountain View, CA, USA) was used to determine the pharmacokinetic parameters of ARI and D-ARI, for each rat, by non-compartmental analysis. The elimination rate constant (K_el_) was calculated by log-linear regression of each plasma concentration–time curve during the elimination phase, with the terminal half-life (T_1/2_) calculated as 0.693/K_el_. The area under the concentration–time curve from the administration of ARI to the last measured concentration at 30 h post-administration (AUC_0–30_) was determined using linear trapezoidal integration. The residual AUC, calculated as the last non-zero concentration in each animal divided by K_el_, was added to AUC_0–30_ to obtain the total AUC (AUC_0–∞_). Other determined parameters were C_max_, the time to reach C_max_ (T_max_), mean residence time (MRT), apparent volume of distribution (Vd/F) and apparent total clearance (CL/F). The metabolite-to-parent ratio (MPR) was calculated using the AUC_0–∞_ for each rat and then averaged for each experimental group. The sum of the AUCs of ARI and D-ARI was calculated for each animal to obtain the individual exposure to the active moiety. Statistical analysis was conducted using IBM SPSS Statistics 30.0.0.0 (Chicago, IL, USA). The normality of data distribution was evaluated using the Shapiro–Wilk test. For parameters meeting normality assumptions, comparisons between reference and test groups were performed with a two-tailed independent t-test. For parameters that did not meet normality assumptions, the nonparametric Mann–Whitney U test was applied, as it is appropriate for comparing two independent groups with continuous variables in relatively small samples, without assuming normality. Statistical significance was set at *p* < 0.05.

### 2.6. Ethical Considerations

The experimental protocol was approved by the Ethics Committee of the “Iuliu Hațieganu” University of Medicine and Pharmacy and by the competent authority, approval number: 322/02.08.2022. The protocol adheres to the 43/2014 Law on animal protection used for scientific purposes, as published in the Official Gazette (Monitorul Oficial) of Romania, which transposes Directive 2010/63/EU of the European Parliament and Council on the protection of animals used for scientific purposes.

## 3. Results and Discussion

**Figure 1:**
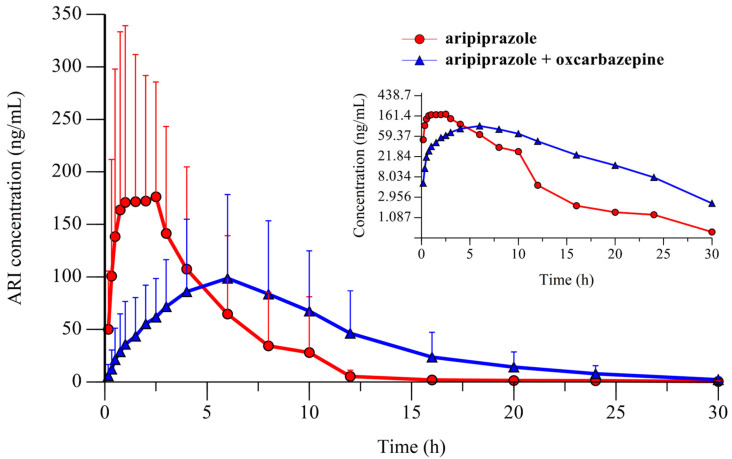
The mean plasma concentration profile of aripiprazole after a single oral dose of 8 mg/kg (*n* = 12, ○) and following a five-day oral oxcarbazepine 85 mg/kg pretreatment (*n* = 12, △). Insert: semi-logarithmic plot.

**Figure 2:**
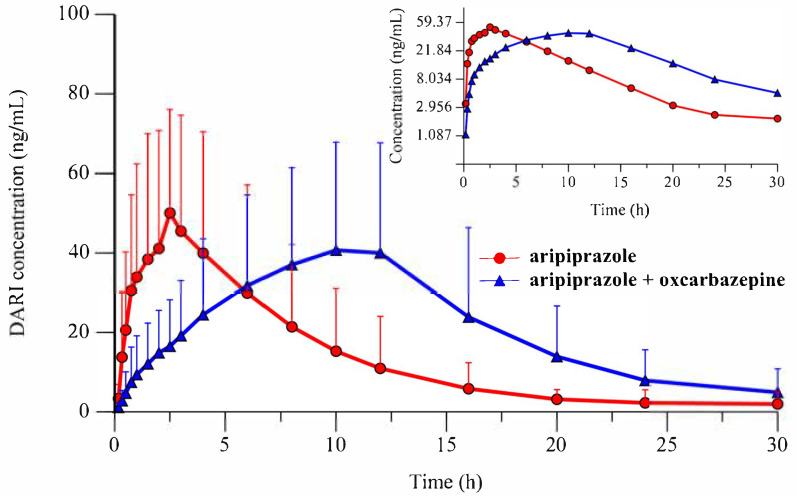
The mean plasma concentration profile of dehydroaripiprazole after a single oral dose of 8 mg/kg aripiprazole (*n* = 12, ○) and following a five-day pretreatment with oral oxcarbazepine 85 mg/kg (*n* = 12, △). Insert: semi-logarithmic plot.

The mean pharmacokinetic parameters (±S.D.) of ARI and D-ARI are summarized in Table 1 and Table 2. Figure 1 shows the mean plasma concentration–time profiles of ARI administered alone as a single dose and after OXC pretreatment. The mean plasma concentration–time profiles for D-ARI from the two experimental groups are shown in Figure 2.

The MPR was 0.43 ± 0.18 in the control group and 0.63 ± 0.16 in the test group. The mean AUC_0–∞_ of the active moiety ± SD was 1578.46 ± 1217.883 h·ng/mL for the reference group and 1762.815 ± 1086.678 h·ng/mL for the test group (*p* > 0.05).

Comedication with CYP3A4 inducers can alter plasma levels of ARI [17]. Psychiatric patients are often polymedicated and present with multiple comorbidities. For instance, epilepsy occurs 4–5 times more frequently among individuals with schizophrenia than in the general population [11]. Additionally, patients with schizophrenia often suffer from focal epilepsy (formerly known as partial-onset epilepsy) [11], for which OXC is a first-line therapeutic option. Therefore, the concomitant use of ARI and OXC is likely and requires investigation because of the potential for pharmacokinetic interaction that may reduce the systemic exposure and efficacy of ARI.

This study is the first to analyze the pharmacokinetic influence of OXC on single-dose ARI. The mean C_max_ of ARI decreased by 2.05-fold following the administration of OXC at a dose of 85 mg/kg (239.67 ± 168.59 vs. 116.55 ± 70.33 ng/mL). Conversely, the mean K_el_ value increased by 1.4-fold, leading to a 1.51-fold decrease in the mean T_1/2_ of ARI (5.56 ± 3.13 vs. 3.69 ± 1.30 h). For the metabolite D-ARI, although not statistically significant, the mean AUC_0–30_ value increased by 38.64%, and the mean AUC_0–∞_ value increased by 44.66%. The mean K_el_ decreased by 25%, resulting in a 26.16% increase in the mean T_1/2_ after OXC pretreatment.

Given the limited presystemic (first-pass) metabolism of ARI, due to its high oral bioavailability, it is likely that the changes in its pharmacokinetic parameters are primarily due to the accelerated systemic metabolism of ARI mediated by the enzyme-inducing effect of OXC on the CYP3A4 isoenzyme at the hepatic level. Through its active metabolite licarbazepine, OXC is considered a mild-to-moderate, dose-dependent inducer of CYP3A4, with no reported influence on CYP2D6 [18,19,20]. ARI is a substrate for both CYP2D6 and CYP3A4, with CYP3A4 playing a more prominent role in the formation of D-ARI [2,3,5,21]. Accordingly, the changes observed in certain pharmacokinetic parameters, like the decrease in mean C_max_ and T_1/2_, together with the slight and statistically non-significant increase in D-ARI’s AUC, may be attributed to enhanced metabolism of ARI via the CYP3A4 pathway, mediated by OXC induction. Additionally, we found no conclusive evidence that OXC significantly alters gastric motility. While nausea and vomiting are the most commonly reported gastrointestinal adverse reactions, both diarrhea and constipation have been reported [7]. As such, any impact on ARI absorption via altered gastrointestinal transit remains inconclusive.

Although induction of metabolizing enzymes may accelerate the biotransformation of ARI to D-ARI, compensatory processes in absorption and distribution can preserve overall exposure, resulting in relatively unchanged AUCs despite significant changes in ARI’s C_max_, T_max_ and K_el_. Moreover, the large SDs highlight considerable inter-individual variability, which can obscure meaningful individual changes in AUC, making differences between experimental groups statistically non-significant. The same inter-individual variability was also evident for CL/F and Vd/F, in addition to the fact that these two parameters were calculated as apparent parameters following oral administration and therefore incorporate the fraction of bioavailability. In contrast, individual T_1/2_ values were derived from individual K_el_s obtained from the terminal slope of the plasma concentration–time curves. As a result, changes in the terminal elimination phase may shorten T_1/2_ even when CL/F and Vd/F do not differ significantly. Furthermore, because in non-compartmental analysis CL/F and Vd/F cannot distinguish between absorption-related changes in bioavailability and true systemic clearance (which includes possible metabolic induction), any observed differences may reflect contributions from either process.

While D-ARI does not have identical activity to ARI, its pharmacological properties are sufficiently similar, such that the sum of the parent drug and its metabolite is regarded as the active moiety. In our study, the mean active moiety AUC increased by 11.68% (*p* > 0.05), indicating that, overall, pharmacologically active exposure was maintained. The observed increase in the mean MPR in the test group compared with the control group is consistent with the inductive effect of OXC on CYP3A4. This increase was likely due to OXC’s inductive effect on CYP3A4, leading to enhanced ARI metabolism and D-ARI production. These results suggest that prior or concomitant treatment with OXC may reduce the body exposure to ARI, enhance its apparent systemic clearance and consequently potentially diminish its clinical efficacy.

Some case reports described a presumed effect of concomitant OXC on the efficacy and exposure of ARI. In a 12-year-old epileptic patient with psychotic symptoms, ARI 15 mg/day was administered together with OXC. Before the treatment regimen was changed, plasma concentrations of ARI and D-ARI were 42 and 19 µg/mL, respectively, well below the adult reference ranges of 120–270 ng/mL for ARI and 180–380 ng/mL for its active moiety [22], according to the most up-to-date research insights [23]. Another case described a 10-year-old boy with aggressive behavior treated with OXC 1200 mg/day and ARI 20 mg/day. At steady state, trough serum concentrations of ARI and D-ARI were 99.8 ng/mL and 53.5 ng/mL, and the C/D ratios were reduced by 68% and 50% [24]. These changes were attributed to the enzyme-inducing effects of OXC on ARI metabolism. Previous clinical trials and therapeutic drug monitoring studies have shown that strong CYP3A4 inducers, such as carbamazepine [25,26], can significantly reduce ARI plasma concentrations and may compromise its clinical efficacy. Valproic acid has also been reported to alter ARI levels [26].

As for the animal model used in the experiment, the in vivo rat model is often employed to study pharmacokinetic interactions, including metabolizing-enzyme-inducing drug–drug interactions [16,27,28]. Rats share physiological similarities with humans, such as a homologous CYP3A4 gene, although not identical, which makes them useful for predicting effects in humans [29]. However, any differences in CYP3A isoforms, even single amino acid substitutions, can modify enzyme activity and substrate specificity [30]. Moreover, extrapolation of doses from rats to humans is inherently limited, as scaling across species does not always account for differences in metabolic capacity. Thus, results from rat studies should be interpreted with caution and supplemented by studies in humans.

For patients with concomitant CYP3A4 perpetrators and substrates, close therapeutic drug monitoring, when available, remains the best option compared with making arbitrary, uniform dose corrections. Our in vivo rat data showed considerable variability in ARI exposure when co-administered with OXC, suggesting that the magnitude of the interaction may differ across individuals. Taken together with the limited human evidence available, specifically two case reports describing reduced ARI efficacy in pediatric patients treated concomitantly with OXC, these observations indicate that reduced drug exposure, with suboptimal efficacy, may occur in a subset of patients due to this interaction, although not all individuals are necessarily affected.

## 4. Conclusions

A five-day pretreatment with oxcarbazepine decreased the mean peak plasma concentration of the aripiprazole parent compound, administered as a single dose, by twofold and shortened its mean half-life by 1.51-fold. The concomitant increase in the metabolite’s area under the curve, although not statistically significant, resulted in a relatively unchanged total AUC of the active moiety. This indicates that the impact of oxcarbazepine was not evident on the overall exposure of the pharmacologically active fraction. Nevertheless, given the need for frequent co-prescription of antiseizure and antipsychotic drugs, it remains important for healthcare providers to monitor changes in aripiprazole exposure to ensure the reliability of its therapeutic effect. Inter-individual variability in CYP3A4 activity may lead to different concentration profiles across patients, making some individuals more sensitive to this drug–drug interaction.

## Figures and Tables

**Table 1:** Pharmacokinetic parameters (mean ± S.D.) for aripiprazole after the administration of a single oral dose of 8 mg/kg and following a five-day oral oxcarbazepine 85 mg/kg pretreatment.

Pharmacokinetic Parameters (U.M)	Aripiprazole Group (Reference, *n* = 12)	Aripiprazole + Oxcarbazepine (Test, *n* = 12)	*p*-Value
C_max_ (ng/mL)	239.67 ± 168.59	116.55 ± 70.33	0.0291 ^a^*
T_max_ (h)	1.65 ± 0.80	4.29 ± 2.41	0.0015 ^a^*
AUC_0–30_ (h·ng/mL)	1109.98 ± 931.46	1080.29 ± 673.92	0.9295 ^a^
AUC_0–∞_ (h·ng/mL)	1117.34 ± 931.41	1095.71 ± 679.74	0.9488 ^a^
K_el_ (1/h)	0.15 ± 0.06	0.21 ± 0.06	0.0424 ^a^*
T_1/2_ (h)	5.56 ± 3.13	3.69 ± 1.30	0.0466 ^b^*
MRT (h)	4.63 ± 1.78	8.98 ± 2.52	<0.0001 ^a^*
CL/F (L/h/kg)	14.86 ± 15.8	10.06 ± 5.53	0.7490 ^b^
Vd/F (L/kg)	121.64 ± 143.94	49.75 ± 24.80	0.1936 ^b^

* Statistically significant, *p* < 0.05; ^a^ *p*-values calculated using a two-tailed independent *t*-test; ^b^ *p*-values calculated using Mann–Whitney U test.

**Table 2:** Pharmacokinetic parameters (mean ± S.D.) for dehydroaripiprazole after the administration of a single oral dose of aripiprazole 8 mg/kg and following a five-day pretreatment with oral oxcarbazepine 85 mg/kg.

Pharmacokinetic Parameters (U.M)	Aripiprazole Group (Reference, *n* = 12)	Aripiprazole + Oxcarbazepine (Test, *n* = 12)	*p*-Value
C_max_ (ng/mL)	57.68 ± 31.31	44.96 ± 25.19	0.2847 ^a^
T_max_ (h)	2.25 ± 1.06	9.58 ± 3.00	<0.0001 ^b^*
AUC_0–30_ (h·ng/mL)	435.81 ± 313.12	605.12 ± 378.75	0.2380 ^b^
AUC_0–∞_ (h·ng/mL)	461.13 ± 339.82	667.10 ± 430.54	0.2150 ^b^
K_el_ (1/h)	0.16 ± 0.11	0.12 ± 0.05	0.3843 ^b^
T_1/2_ (h)	5.58 ± 2.89	7.04 ± 3.89	0.3735 ^b^
MRT (h)	7.61 ± 3.48	14.1 ± 4.64	0.0009 ^b^*
CL/F (L/h/kg)	41.64 ± 55.66	15.89 ± 7.45	0.2150 ^b^
VdF (L/kg)	238.38 ± 225.81	169.07 ± 150.70	0.2846 ^b^

* Statistically significant, *p* < 0.05, ^a^ *p*-values calculated using a two-tailed independent *t*-test, ^b^ *p*-values calculated using Mann–Whitney U test.

## Data Availability

The original raw data underlying plasma concentrations and individual pharmacokinetic parameters generated in this study are openly available in Zenodo at https://doi.org/10.5281/zenodo.17850660. Further inquiries can be directed to the corresponding author.

## Abbreviations

The following abbreviations are used in this manuscript:

ARI	Aripiprazole
AM	Active Moiety
D-ARI	Dehydroaripiprazole
OXC	Oxcarbazepine
LS-MS/MS	Liquid Chromatography
HPLC	High Performance Liquid Chromatography
C_max_	Peak Plasma Concentration
T_max_	Time to Reach the Maximum Plasma Concentration
AUC_0–30_	Area Under the Plasma Concentration–Time Curve from Time Zero to 30 Hours
AUC_0–∞_	Total Area Under the Curve
K_el_	Elimination Rate Constant
T_1/2_	Half-Life
MRT	Mean Residence Time
CL/F	Apparent Systemic Clearance
Vd/F	Apparent Volume of Distribution
CYP	Cytochrome P450
b.w.	Body Weight
rpm	Rotations per Minute
MPR	Metabolite-to-Parent Ratio
